# Management Challenges in Chronic Obstructive Pulmonary Disease in the COVID-19 Pandemic: Telehealth and Virtual Reality

**DOI:** 10.3390/jcm10061261

**Published:** 2021-03-18

**Authors:** Sebastian Rutkowski

**Affiliations:** Department of Physical Education and Physiotherapy, Opole University of Technology, 45-758 Opole, Poland; s.rutkowski@po.opole.pl; Tel.: +48-507-027-792

**Keywords:** COPD, COVID-19, pulmonary rehabilitation, virtual reality, telemedicine

## Abstract

For the treatment of chronic obstructive pulmonary disease (COPD), early diagnosis and unconditionally correct management at the initial stage of the disease are very important when the symptoms are not yet too worrying. In this way, the progress of the disease can be slowed down, as can the occurrence of late, life-threatening symptoms. Pulmonary rehabilitation is an essential component of the management of COPD. The selection of appropriate exercises, which are determined during the classification of patients into a suitable improvement program, is of key importance in the process of rehabilitation. The coronavirus disease 2019 (COVID-19) pandemic has resulted in major limitations to public health care. Health systems were largely unprepared for an outbreak of this magnitude. Searching for new, attractive technologies that help patients with chronic diseases seems to be justified. This may be driven by telehealth platforms, likewise with the use of virtual reality (VR). Analysis of the available literature indicates promising effectiveness, high patient acceptance, and high motivations to undertake physical activity with the use of such a solution. Thus, the management of patients with COPD during the COVID-19 pandemic should include options for remote delivery of pulmonary rehabilitation, including home-based, telerehabilitation, and computer-based virtual programs.

## 1. Introduction

Coronavirus disease 2019 (COVID-19), which is caused by severe acute respiratory syndrome coronavirus 2 (SARS-CoV-2), appeared for the first time in Wuhan, Hubei Province, China, in early December 2019. On 31 December 2019, an outbreak of atypical pneumonia was announced in Wuhan [1,2]. The COVID-19 infection has quickly spread to many other countries around the world, triggering the reaction of the World Health Organization (WHO), which on 11 March 2020 declared a pandemic status.

Over recent months, SARS-CoV-2 infection has been confirmed in millions of people around the world. Clinical symptoms of patients with COVID-19 infection include fever, sore throat, coughing, fatigue, or gastrointestinal infections, which can appear in a smaller population of patients. In more severe cases, respiratory failure symptoms, as well as heart and kidney damage, may occur. This can happen especially in the elderly and in people with other concomitant chronic diseases [3]. About 60 days after onset of the first COVID-19 symptom, only 13% of the patients previously hospitalized for COVID-19 were completely free of any COVID-19-related symptoms, while 32% had one or two symptoms and 55% had three or more symptoms [4]. Goërtz et al. assessed multiple relevant symptoms recovery following the onset of symptoms in hospitalized and non-hospitalized patients with COVID-19. Fatigue and dyspnoea were the most prevalent symptoms during the infection and at follow-up (fatigue: 95% versus 87%; dyspnoea: 90% versus 71%). It was concluded that there was only a partial recovery in symptoms about 3 months after the onset of symptoms in a survey of a large sample of previously hospitalized and non-hospitalized patients with confirmed or suspected COVID-19. Survivors of severe COVID-19 are significantly impaired in all activities of daily living and are in need of multimodal rehabilitation, with particular knowledge in cardiovascular and pulmonary medicine [5]. It is highly anticipated that some patients with COVID-19 will have a need for rehabilitation interventions during and immediately after hospitalization [6,7]. It is assumed that many rehabilitation programs for this patient group will be based on pulmonary rehabilitation programs.

Thus, the purpose of this review was to assess the challenges that the COVID-19 outbreak has on the management of chronic obstructive pulmonary disease (COPD). It would seem that identifying the potential for the use of new technologies, which can be highly applied remotely, may contribute to the optimizing of the health care system for broad availability of pulmonary rehabilitation. In the opinion of the author, it remains apparent that despite widely available evidence, the use of technology is being downplayed, and the belief that only face-to-face therapy can provide benefits is misguided.

## 2. An Overview on COPD Management

Chronic obstructive pulmonary disease (COPD) is a major threat to public health and is recognized as one of the most impactful common chronic diseases, making it the second most common cause of disability [8]. For the treatment of COPD, early diagnosis and unconditionally correct management at the initial stage of the disease are very important when the symptoms are not yet too worrying. In this way, the progress of the disease can be slowed down and the occurrence of late, life-threatening symptoms like dyspnea, reduced exercise tolerance, and finally respiratory disability can be delayed; these symptoms significantly reduce the quality of life of patients. A “stable” COPD condition has been defined when symptoms are well controlled and deterioration of lung function is minimized, whereas managing “unstable” COPD (when patients have frequent or severe exacerbations) can be more difficult. COPD exacerbations are a major economic burden and can result in emergency department admission and hospitalizations [9]. Severe exacerbations are associated with a significant increase in mortality, therefore prevention of exacerbations is one of the key goals in the management of COPD [10]. The remaining key issues include assessment of the severity of airflow obstruction, exposure of risk factors, and comorbidities, as well as other symptoms. Treatment can be escalated/de-escalated based on the presence of the predominant symptoms of breathlessness and exercise limitation, and the continued occurrence of exacerbations whilst on maintenance therapy [10]. An obligatory element of the elimination of risk factors by patients is smoking cessation. Initial pharmacotherapy should be based on the patient’s Global Initiative for Obstructive Lung Disease (GOLD) group. Due to the chronic nature of the disease, patients should be educated in self-management at the initial stage. This should include risk factor management, inhaler technique, breathlessness, and a written action plan. Patient awareness of current symptom levels (either the CAT or mMRC scores) and exacerbation frequency assessments have also been found to be very important. Thus, the existing management models for patients with COPD have assumed pharmacological-oriented management for “unstable” COPD and non-pharmacological (rehabilitation-oriented) management for stable COPD. The Task Force co-chairs (J.A. Wedzicha and J.A. Krishnan) were selected by the European Respiratory Society and American Thoracic Society, established to develop recommendations for prevention of exacerbations [11]. The Task Force made recommendations for mucolytic, long-acting muscarinic antagonist, phosphodiesterase-4 inhibitor (roflumilast), and macrolide therapy, as well as a conditional recommendation against fluoroquinolone therapy. Thus, it seems that the management of patients with unstable COPD lies with the physicians who provide a treatment and action plan for dealing with exacerbations. Therefore, a form of telemonitoring and teleconsultation may have a practical application in this regard. However, there is much more adaptability in the management approach of stable COPD. Pulmonary rehabilitation (PR) is an essential component of the management of COPD. The selection of appropriate exercises, which are determined during the classification of patients into a suitable improvement program, is of key importance in the process of rehabilitation. The adopted models of PR vary in terms of intensity, duration, and the form of physical activity taken by the patients. Many research studies and systematic literature reviews show the beneficial effect of PR in patients with chronic respiratory diseases on exercise capacity [12], lung function [13], respiratory muscle strength [14], and quality of life [15]. The effects of PR programs are described in detail in the available literature. There are probably already enough observational studies linking physical inactivity with poor outcomes in COPD. However, increasing exercise tolerance facilitates, but does not necessarily yield, increases in physical activity. Troosters et al. had compared effects on the exercise tolerance benefit of a bronchodilator (19 studies, 1847 individuals) vs. PR (11 studies, 559 individuals) showed 3 times more effectiveness of PR programs on increases in endurance time in constant work rate exercise testing [16]. However, international estimates posit that only 1–2% of COPD patients receive PR. In contrast, other COPD therapies, bronchodilators and oxygen therapy in particular, are much more widely available [17]. With the already low availability of rehabilitation programs, the current situation makes this aspect even worse. Therefore, as a next step, it was decided to assess the impact of the pandemic on the healthcare modalities as well as to propose possible solutions, re-orienting the focus on the use of new technologies.

## 3. Management of COPD’s Patients in the COVID-19 Pandemic

The COVID-19 pandemic has resulted in major limitations to public health care. The healthcare systems were highly unprepared for an outbreak of this magnitude. The impact of the COVID-19 pandemic on health systems resilience was the subject of an Organization for Economic Co-operation and Development (OECD) and WHO report. Prominent crisis response measures included securing medical supply chains, ensuring the availability of health workers, mobilizing additional financing, reorganizing non-COVID-19 related health services, using digital solutions to monitor and manage COVID-19 cases as well as providing medical services online (e.g., teleconsultations). Many governments and information services have developed mHealth initiatives. The humanitarian and economic demands of COVID-19 have been driving both the rise and adoption of new digital technologies. The pandemic has raised many issues regarding the management of patients with COPD and the appropriateness of modifying their therapy. Questions have arisen about recognizing and differentiating COVID-19 from COPD because of the similarity of symptoms [18]. It is unclear whether patients with COPD are at increased risk of becoming infected with SARS-CoV-2. However, it is worth noting that some of the elements related to the management of patients with COPD can be conducted remotely, which may reduce the risk of potential infection.

### 3.1. Telehealth 

Remote care or telehealth services application has accelerated. Telehealth services have now been used in the large-scale screening of patients, for remote clinical encounters, or supervising patient care by experts [19,20]. Telehealth provides higher convenience and a better patient-centered approach, helping to improve the flow of healthcare systems [21]. Telehealth refers to the delivery of different health care services for patients through the technologies of telecommunication, and includes (1) telemedicine and (2) telehealthcare. The telehealthcare area covers: telehomecare, telenursing, telecoaching, and telerehabilitation. Meanwhile, telemedicine can be applied with the help of teleservices in the field of: teleradiology, teledermatology, telepsychiatrists, telecardiology, and telerehabilitation. Telemedicine can be defined, according to the European Commission, as “the provision of healthcare services, through the use of ICT, in situations where the health professional and the patient (or two health professionals) are not in the same location. It can be utilized to securely transmit medical data necessary for the prevention, diagnosis, treatment, and follow-up of patients” [22]. In 2002, an official telerehabilitation was recognized to provide access to a collection of rehabilitation services for the disabled “at a distance” through telecommunication sites and the Internet. Nowadays, with a computer, webcam, and appropriate software, it is possible to create a videoconference enabling the physiotherapist to give advice and practice at a distance. The systematic reviews suggest that telerehabilitation is becoming increasingly popular due to the need to provide equitable access to rehabilitation for populations with barriers to accessing traditional models of care [23], likewise it can be considered as an alternative approaches to reduce outpatient resource utilization and improve quality of life [24].

The telehealth services in pulmonary rehabilitation have been successfully carried out so far via telemonitoring and telerehabilitation [25,26,27]. Home telehealth services in this field were found to reduce rates of hospitalization and emergency department visits. Most studies found home telehealth to be cost-saving from healthcare system and insurance provider perspectives [28], and to be equally effective for hospital-based outpatients [29]. The telerehabilitation program was proven to be particularly effective in monitoring disease exacerbations. This has been driven by two activities: (1) telespirometry for remote measurement of lung function, initially to diagnose COPD and periodically to ascertain clinical status; and (2) teleconsultations between primary care providers (physicians and nurses) and pulmonary specialists for the care and treatment of patients at remote sites [27]. With regard to the present situation, the advisability of teleconsultation seems particularly important, as it provides access to expert consultants in areas lacking these resources and also reduces unnecessary hospitalization. Moreover, it have been also stated that during periods of high community prevalence of COVID-19, spirometry should only be used when it is essential for COPD diagnosis and/or to assess lung function status for interventional procedures or surgery [18]. Therefore, the use of remote diagnostic techniques appears to be of great practical importance. Data analysis of the study of 937 Italian general physicians who, over a 2-year period, received the results of 20,757 telespirometries tests, revealed that 70% of the tests met the criteria for good or partial co-operation, allowing spirometric abnormalities to be detected in more than 40% of the tracings [30]. Vitacca et al. investigated the change in the patient population, mortality, and staff utilization/costs during the first 5-year activity of a teleassistance program. The results showed a shift in costs, with an overall decrease in physician time and an increase in nurse time, thereby resulting in cost savings of 39% [31]. Sorknaes et al. investigated the effect of daily teleconsultations for one week between in-hospital nurses and patients with severe COPD discharged after an acute exacerbation. Teleconferences were conducted via computer with a web camera and a microphone along with measurement equipment for about 7 days. A total of 266 patients were allocated to either intervention (*n* = 132) or control (*n* = 134). Data analysis showed no significant difference in the unconditional total mean number of hospital readmissions after 26 weeks or effects on mortality [32]. Another multicenter randomized controlled trial study was investigated by Pinnock et al. on a sample of 256 COPD patients in the UK. The intervention consisted of daily patient responses entered on a touchscreen device or conventional self-monitoring to questions about symptoms and the use of treatment and oxygen saturation. The authors stated that in participants with a history of admission for exacerbations of COPD, telemonitoring was not effective in postponing admissions and did not improve quality of life [33]. In contrast, Pare et al. found reductions in home visits and hospitalizations in patients with COPD who were treated with the telehomecare model and significant cost savings after 6 months of follow-up [34]. Alrajab et al. likewise reported a reduction in the frequency of COPD exacerbations with the use of a telemonitoring program [35]. Cordova et al. in their randomized study showed that a telemedicine-based symptom reporting program facilitated early symptom management and improved lung function and functional status in patients previously hospitalized for exacerbations. Thus, it seems that the use of telemonitoring systems for patients with COPD may have practical applications in unstable COPD. However, it is important to note that some studies appear to present small sample sizes and limited follow-up time as factors that must be addressed before telemedicine interventions can be considered as the standard of care for COPD patients.

Regarding the telerehabilitation interventions, the recent literature has suggested that telerehab is as effective as in-hospital PR programs, and confirmed its safety and feasibility [36,37,38]. Advantages of telerehabilitation include a reduced caregiver burden, lower costs, adherence to recommendations, exercise progression, home environment, and objective physical activity monitoring [39]. The major benefits were demonstrated in reduced dyspnea, improved functional capacity, quality of life, and high adherence to the exercise programs. Similar to traditional PR, implementation of telerehabilitation leads to improvement in dyspnea, exercise capacity, and lung function, as well as reductions in hospitalization and mortality [40,41,42]. The programs were mostly based on videoconferencing (online face-to-face) or phone calls to run supervised training using performance training equipment (treadmill or cycling) and/or free exercise (fitness-, aerobic-, strength-, yoga- training) [43,44,45]. Therapies were delivered to patients in moderate to severe stages of disease. The second group of interventions consists of unsupervised trainings through the use of a virtual trainer or pre-recorded video demonstrations. The studies by Bourne et al. [44] and Chaplin et al. [46] compared the effect of unsupervised web-based or video-demonstrated individual exercise with the conventional group PR. They found comparable effects on walking tests that exceeded the minimal clinically important difference for 6MWD and ESWT but not for ISWT. There is an ever-growing and complex body of empirical evidence that attests to the potential of telemedicine for addressing problems of access to care, quality of care, and healthcare costs in the management of pulmonary chronic diseases [27]. Recently, the focus has shifted towards unsupervised or AI-supervised (workloads planned based on initial qualification) training programs. This, opens up possibilities for the use of virtual reality (VR) for patients with pulmonary disease.

### 3.2. Virtual Reality

Virtual Reality is the technology that provides almost real and/or believable experiences in a synthetic or virtual way. In practice, virtual reality is a combination of specialized hardware and software [47]. This technology continues to develop rapidly and gain a growing number of enthusiasts in different age categories. It is most often associated with entertainment, but it applies much more widely in other fields. The literature describes the use of the so-called “Virtual Rehabilitation” in the therapy of patients, using the audiovisual biofeedback system [48]. The literature contains publications describing the possibility of using VR as a diagnostic and therapeutic tool. The majority of the research on virtual rehabilitation focuses on neurorehabilitation. However, interest in this technology increases and extends to orthopedic, pediatric, and psychiatric oncology as well as pulmonary rehabilitation [49]. Jacobson described four types of VR: immersive virtual reality, desktop virtual reality (i.e., low-cost game console) also called a non-immersive, augmented virtual reality (where computer-generated data merge into a real-world image), and simulation (mixed) virtual reality (a combination of real objects and environments with virtual people or places, either controlled by humans or by artificial intelligence) [50]. The essence of immersion seems to presents a great practical significance in pulmonary rehabilitation, where the patient can become more involved. Building involving scenarios can facilitate a “shift in attention,” distracting the patient from negative sensations (e.g., fatigue, dyspnea) during physical activity [51]. Moreover, it has been shown that the use of virtual reality in the rehabilitation process changes patient engagement in therapy [52]. Patients become motivated to exercise by having the opportunity to experience an interesting world, in contrast to monotonous workouts. This component seems to be crucial in the case of chronic patients such as COPD patients. It is well known that the basis of rehabilitation in this group of patients lies in endurance training, which often accompanies patients throughout their lives in order to prevent disease progression. Thus, the use of an attractive, engaging approach seems justified. The use of mixed virtual reality, on the other hand, makes it possible to generate a projection/image of the trainer which can be used as a source of free exercises, like group gymnastics, where the instructor demonstrates a movement, which the patients should copy [53]. Yet, the application of virtual reality with pulmonary rehabilitation is limited. The latest systematic review and meta-analysis of the effectiveness of virtual reality for patients with the respiratory disease was published in November 2020. Twenty-two articles were analyzed: seven were identified as pilot or feasibility studies, four as systematic reviews, six as randomized controlled trials, and five as observational studies. Six studies recruited individuals with CF, one article analyzed asthma, and four articles analyzed subjects with COPD. The benefits of interventions delivered in VR have been categorized into several aspects. The authors stated that exergaming-based interventions compared with conventional PR demonstrated an insignificant effect (SMD −0.17 [−0.43–0.09]) on mean HR. Next, a weak effect was found of SpO2 (SMD 0.22, 95% [CI, −0.25–0.69]), as VR interventions resulted in a lower level of oxygen depletion. Furthermore, VR interventions induced a lower level of dyspnea [54]. The previous meta-analysis of Wang et al. investigated the effectiveness of active video games for patients with COPD [55]. Analysis of results showed that less technologically-advanced game consoles used in addition to PR can be useful and enjoyable. However, this review included only 7 studies (3 RCT), suggesting the need for further development of this technology. A technological analysis of VR systems was also attempted. Colombo et al. analyzed a technological perspective of VR for COPD rehabilitation [56]. In the first part of the study, the team conducted a scoping review, identifying 21 articles: 10 journal papers, five conference proceedings papers, five abstracts, and one book chapter. The authors noted that most of these studies (68%) used active video games as an intervention for physical and breathing exercises, six systems considered VR systems, and two studies provided intervention through mobile applications. Analysis of the effectiveness of such systems has shown. Rehabilitation programs varied in duration (2 to 8 weeks) and the evaluated parameters (mainly focused on physical training). The reviewed papers suggested that VR-based training is feasible for patients with COPD. Authors concluded that further research should focus on physiological interaction during such training to provide more objective data.

The possibility of implementing VR for the rehabilitation of patients with COPD is also of interest to the authors personally. Among the first clinical experiments, effectiveness of fitness using non-immersive VR training was analyzed. The study consisted of 68 patients with COPD to a 2-week, five times a week in-hospital PR [57]. This appears to be the first study to evaluate the effectiveness of VR system implementation in patients with moderate to severe COPD undergoing an intensive 2-week inpatient rehabilitation program. Analysis of the results showed that patients who participated in the VR sessions achieved significant improvement in physical fitness in all attempts of the Senior Fitness Test. The second clinical experiment conducted under the scientific supervision of Professor Richard Casaburi aimed to assess the influence of immersive VR on the exercise responses during a submaximal exercise test (ET) on a cycle ergometer, with a future goal to implement this type of test for patients with COPD. The study enrolled 70 healthy volunteers. Each participant performed an ET with and without VR. Both tests consisted of incrementing the work rate every 3 min and were terminated when the subject reached 85% of the predicted heart rate (HR). VR was created with an HTC Vive Pro head-mounted display, along with VR healthcare (aerobic exercise) cycling software. Data analysis showed that HR was consistently lower in the VR tests over a wide range of work rates, particularly within the first 3 min of testing, and likewise in the last 3 min of testing. Moreover, there were distinct differences in the number of HRV variables between the two modes of testing. In the initial phase of the exercise, the tests with VR compared to those without VR demonstrated significantly higher values for all HRV parameters (except LF/HF) (i.e., SDNN, RMSSD, TP, LF, and HF). Since these parameters as a majority reflect the parasympathetic activity, it is apparent that VR activated the parasympathetic portion of the autonomic nervous system, which also yielded lower values of HR throughout the exercise; consequently, the submaximal exercise lasted longer. This confirms the hypothesis that therapy conducted in VR is safer for the cardiovascular system compared to that conducted in the traditional form [58]. Furthermore, the STIIMA research group carried out, in cooperation with the IRCCS INRCA (an Italian clinical center specialized in the rehabilitation of elderly with respiratory diseases), a clinical study evaluating the effectiveness of a VR-based pulmonary rehabilitation program in a group of patients with COPD. Preliminary results showed that an endurance training program based on semi-immersive (i.e., wide projected screen) VR, called the “Virtual Park”, is feasible, safe, and well accepted by patients, who felt more motivated when following the prescribed cycling protocol [59]. The third clinical experiment aimed to evaluate the efficacy of VR therapy on depressive and anxiety symptoms and stress levels in patients with COPD during in-hospital pulmonary rehabilitation [60]. The study consisted of 50 patients diagnosed as having COPD, who were randomly assigned to one of two groups. Both participated in the traditional PR program, whereas the VR group completed 10 sessions of immersive VR therapy, while the control group engaged in 10 sessions of Schultz Autogenic Training. As a VR source, the VR Tier One device (Stolgraf^®^) was used. The results of the experiment showed a reduction in stress levels only within the VR group. Moreover, a reduction in depressive and anxiety symptoms was also reported only in the VR group for both parameters with a strong effect size. VR therapy was revealed to be more effective than the traditionally used Schultz Autogenic Training to reduce stress, anxiety, and depression symptoms. Among other reports evaluating the effectiveness of VR in patients with COPD, a common conclusion indicated patients’ confidence in the attractive form of exercise, building high motivation to undertake physical activity but also the need for further research on the possibilities of such training [61,62,63].

Thus, it appears that in stable COPD, the use of modern technology in the form of real-time telemedicine interventions or the transfer to computerized programs in the form of virtual trainers or video games is useful. Scientific evidence shows that therapy delivered in a virtual world is highly effective. The virtual world also allows the creation of a new environment for the patient, resulting in increased patient engagement, which can lead to behavioral changes and therefore to increased levels of physical activity. Also, it may be important in the situation of further lockdowns to have an alternative to in-hospital rehabilitation programs to combat the long-term effects of interrupted training.

## 4. Conclusions

The “Global Initiative for Obstructive Lung Disease (GOLD)” included PR in the standard treatment of COPD patients in 2001 [64]. The main goal of pulmonary rehabilitation is to improve the patient’s psycho-somatic state, as well as to facilitate the patient’s return to family and society. Broadly evidenced benefits include improvements in exercise tolerance, muscle strength, lung function, quality of life, likewise reduced dyspnea, hospital admissions, and length of hospital stay [65]. Despite limited access to specialized medical centers, an opportunity for appropriate management of COPD patients with new technologies seems to be available. The three major components of the medical system—prevention, treatment, and rehabilitation—are equally important. This may be driven by telehealth platforms, as well as by the use of VR. Digital communication platforms support adherence to social-distancing measures [66]. Further evidence of the clinical effectiveness of digital technologies being incorporated into public health systems or tested by the National Institute for Health and Care Excellence remains needed [67]. In the opinion of worldwide experts, “novel” forms of pulmonary rehabilitation may be relevant. Professor Richard Casaburi stated: “With the coronavirus disease 2019 crisis, in-center programs have become nearly unavailable. Remote programs are an alternative we have to explore and promote. The debate over whether or not they are equivalent (or even superior) can wait until the pandemic is in the rearview mirror” [68]. Professor believes that the key issue in the future of pulmonary rehabilitation will be its availability: “Improved access will require a fair reimbursement for this therapy by governmental agencies and third-party payors. It will also require training of a new generation of healthcare providers, trained in multiple disciplines, to deliver rehabilitative therapy”. 

Thus, it appears that the management of COPD patients during the COVID-19 epidemic should include remote PR management, including telerehabilitation, and virtual reality platforms, as scientific evidence of their effectiveness becomes more widely available.

## Data Availability

Not applicable.
